# Enhancement of Ischemic Wound Healing by Spheroid Grafting of Human Adipose-Derived Stem Cells Treated with Low-Level Light Irradiation

**DOI:** 10.1371/journal.pone.0122776

**Published:** 2015-06-11

**Authors:** In-Su Park, Phil-Sang Chung, Jin Chul Ahn

**Affiliations:** 1 Beckman Laser Institute Korea, Dankook University, 119 Dandae-ro, Cheonan, Chungnam, 330–714, Korea; 2 Department of Otolaryngology-Head and Neck Surgery, College of Medicine, Dankook University, 119 Dandae-ro, Cheonan, Chungnam, 330–714, Korea; 3 Department of Biomedical Science, Dankook University, Cheonan, Chungnam, 330–714, Korea; 4 Biomedical Translational Research Institute, Dankook University, Cheonan, Chungnam, 330–714, Korea; Massachusetts General Hospital, UNITED STATES

## Abstract

We investigated whether low-level light irradiation prior to transplantation of adipose-derived stromal cell (ASC) spheroids in an animal skin wound model stimulated angiogenesis and tissue regeneration to improve functional recovery of skin tissue. The spheroid, composed of hASCs, was irradiated with low-level light and expressed angiogenic factors, including vascular endothelial growth factor (VEGF), basic fibroblast growth factor (FGF), and hepatocyte growth factor (HGF). Immunochemical staining analysis revealed that the spheroid of the hASCs was CD31^+^, KDR^+^, and CD34^+^. On the other hand, monolayer-cultured hASCs were negative for these markers. PBS, human adipose tissue-derived stromal cells, and the ASC spheroid were transplanted into a wound bed in athymic mice to evaluate the therapeutic effects of the ASC spheroid in vivo. The ASC spheroid transplanted into the wound bed differentiated into endothelial cells and remained differentiated. The density of vascular formations increased as a result of the angiogenic factors released by the wound bed and enhanced tissue regeneration at the lesion site. These results indicate that the transplantation of the ASC spheroid significantly improved functional recovery relative to both ASC transplantation and PBS treatment. These findings suggest that transplantation of an ASC spheroid treated with low-level light may be an effective form of stem cell therapy for treatment of a wound bed.

## Introduction

Formation of new blood vessels, either by angiogenesis or by vasculogenesis, is critical for normal wound healing. Angiogenesis aids in the repair of damaged tissue by regenerating blood vessels and thus improves blood flow in chronic, disease-impaired wounds [1]. To accelerate skin regeneration, many skin tissue engineering techniques have been investigated, including the use of various scaffolds, cells, and growth factors [2]. However, only a subset of the tissue functions can be restored with existing tissue engineering techniques.

Human adipose-derived mesenchymal stem cells (hASCs), which are found in adipose tissue, provide an attractive source of cell therapy for regeneration of damaged skin because they are able to self-renew and are capable of differentiating into various cells [3, 4]. Recent clinical trials involving stem cell therapy aimed to increase vascularization to a sufficient level for wound perfusion and healing [5]. However, several studies claim that the effects of stem cell therapy are not significant in the absence of scaffolds or stimulators [6]. Recently, various scaffolds or growth factors have been studied to increase skin regeneration when using stem cells [7].

Low-level light irradiation (LLLI) has been implemented for various purposes for some time, such as to provide pain relief, to reduce inflammation, and to improve local circulation. Moreover, many studies have demonstrated that LLLI has positive biostimulatory effects on stem cells [8]. For example, LLLT can positively affect hASCs by increasing cellular viability, proliferation and migration [9, 10]; LLLI also enhances vascular endothelial growth factor (VEGF) and fibroblast growth factor (FGF) secretion [8]; and Low-level light therapy (LLLT) enhanced tissue healing by stimulating angiogenesis in various animal models of ischemia [11]. Hypoxic preconditioning results have been reported in enhanced survival of human mesenchymal stem cells [12]. Since cells within a spheroid are naturally exposed to mild hypoxia, they are naturally preconditioned to an ischemic environment [13]. In ischemia models, spheroids of stem cells present improved therapeutic efficacy via enhanced cell viability and paracrine effects [14]. Hypoxia stimulates the production of growth factors, such as VEGF that induce angiogenesis and endothelial cell (EC) survival [13]. In two-dimensional cultures, growth factors secreted from cells are released and diluted into the culture supernatant, preventing cells from responding to the released factors [14].

Several experimental strategies for endothelial differentiation of stem cells have been developed, including 2D-cell culture in EC growth medium containing VEGF and FGF, 3D spheroid culture on substrates with immobilized polypeptides, and genetic modification of stem cells [12, 15, 16]. However, no reports have yet been produced discussing high-ratio EC differentiation of hASCs in 3D-cultured stem cells without growth factors and peptides.

In this study, LLLI was used to promote a hypoxic spheroid of hASCs (which we refer to as a ‘spheroid’) by weakening cell-matrix adhesion. Differentiation and secretion of FGF and VEGF growth factors were also enhanced by LLLI. hASCs can differentiate into ECs without EC growth medium containing VEGF and FGF. The vascularization and potential therapeutic efficacy of ASC spheroids treated with LLLI (L-spheroid) were evaluated by injecting spheroids into a mouse excisional wound splinting model.

## Materials and Methods

### Culture of ASCs

The hASCs were supplied by Cell Engineering for Origin, CEFO (Seoul, Korea) under a material transfer agreement. hASCs were isolated from the adipose tissue and were cultured in low-glucose Dulbecco's modified Eagle's medium F-12 (DMEM/F-12; Welgene, Daegu, Korea) supplemented with 10% fetal bovine serum (FBS, Welgene), 100 units/ml penicillin, and 100 μg/ml streptomycin at 37°C in a 5% CO_2_ incubator. The hASCs between passage 5 and 8 were used for all experiments.

### Spheroid formation

hASCs were split and seeded on 24 well polystyrene plate (low cell binding surface) at a density of 7.5 × 10^4^ cells/cm^2^,andallowedtoadhereat37°C. Within 3 days of culture, hASCs formed spheroids by Low-Level light irradiation (L-spheroids). The light source used was LED (light emitting diode; WON Technology Co., Ltd., korea) designed to fit over a standard multi-well plate (12.5 × 8.5 cm) for cell culture. The LED was had an emission wavelength peaked at 660 nm. The irradiance at the surface of the cell monolayer was measured by a power meter (Orion, Ophir Optronics Ltd., UT). To obtain the energy dose of 6 J/cm^2^,exposure time for LED array was 10 min under power density of 10 mW/cm^2^ (1 milliwatt × second = 0.001 joules) (Table 1). L-spheroid sizes were measured by counting the area of individual cell clusters by image analysis. The diameters of L-spheroids were presented as median ± SD (n = 8 per group).

**Table 1:** Summary table of parameters for the light irradiation.

	*Invitro* study	*Invivo* study
Frequency of irradiation	10 min during 3days	10 min daily from day 1 to 20
Light irradiated site	24 well cell culture plate (12.5 × 8.5 cm)	8-mm (wound site)
Distance from the LED light	10 cm	8 cm
Power density	10 mW/cm^2^	50 mW/cm^2^
Light dosage (Fluence)	6 J/cm^2^	30J/cm^2^
Irradiance (Wavelength)	660 nm	660 nm

### Cells viability assay

After 3 days of culture, the cell viability of the spheroids was analyzed by using a live/dead viability cytotoxicity assay kit (Molecular probes, Carlsbad, CA). Briefly, 1 ml of HEPES-buffered saline solution (HBSS) containing 2 μl of SYTO 10 green fluorescent nucleic acid stain solution and 2 μl of red (ethidium homodimer-2) nucleic acid stain solution were added to plates, and these were then incubated at 37°C in a 5% CO_2_ incubator for 15 min. The negative control was prepared by freezing cells at -80°C for 30 min. Images were quantified by using the ImageJ software (NIH, Bethesda, MD), and the percentage of live/dead cells was scored by counting pixels in each image.

### Fluorescence-activated cell sorting (FACS)

The cells were washed with phosphate buffered saline (PBS) containing 0.5% bovine serum albumin (BSA; Sigma-Aldrich, St. Louis, MO) and were stained in PBS containing 1% BSA, with either isotype controls or antigen specific antibodies, for 60 min. CD34 (BD Biosciences, San Jose, CA), KDR (Beckman Coulter, Brea, CA), CD31 (Beckman Coulter), CD45 (Abcam, Cambridge, MA), CD90 (BD Biosciences), CD105 (Caltac Laboratories, Burlingham, CA), and CD29 (Millipore, Waltham, MA) human antibodies were used. The cells were washed three times with PBS containing 0.5% BSA and were resuspended in PBS for flow cytometry using an Accuri device (BD Biosciences). The isotype IgG was used as a negative control.

### Human angiogenic protein analysis

To analyze the expression profiles of angiogenesis-related proteins, we used a Human Angiogenesis Array Kit (R&D Systems, Ltd., Abingdon, UK). Cell samples (5 × 10^6^ cells) were harvested, and 150 μg of protein were mixed with 15 μl of biotinylated detection antibodies. After pre-treatment, the cocktail was incubated with the array overnight at 4°C on a rocking platform. Following washing to remove unbound material, streptavidin–horseradish and chemiluminescent detection reagents are added sequentially. The signals on the membrane film were detected by scanning on an image reader LAS-3000 (Kodak, Rochester, NY) and were quantified using the MultiGauge 4.0 software (Kodak). The positive signals seen on developed film were identified by placing a transparency overlay on the array image and aligning it with the two pairs of positive control spots in the corners of each array.

### ELISA assay for angiogenic growth factor production

Angiogenic growth factor production in the spheroid was assayed with a commercially available ELISA kit (R&D Systems) according to the manufacturer’s protocols. The concentrations are expressed as the amount of angiogenic growth factor per 10^4^ cells at a given time.

### Immunofluorescence staining

Indirect immunofluorescence staining was performed using a standard procedure. In brief, tissues cryosectioned at a 4-μm thickness were fixed with 4% paraformaldehyde, blocked with 5% BSA/PBS (1 h, 24°C), washed twice with PBS, treated with 0.1% Triton X-100/PBS for 1 min, and washed extensively in PBS. The sections were stained with specific primary antibodies and fluorescent-conjugated secondary antibodies (Table 2) using a M.O.M kit according to the manufacturer’s instructions (Vector Laboratories, Burlingame, CA). The cells were counterstained with DAPI (4,6-diamino-2-phenylindole dihydrochloride; Vector Laboratories). Mouse IgG (Dako, Carpinteria, CA) and rabbit IgG (Dako) antibodies was used as negative controls. To detect transplanted human cells, sections were immunofluorescently stained with anti-human nuclear antigen (HNA, Millipore). The stained sections were viewed with a DXM1200F fluorescence microscope (Nikon, Tokyo, Japan). The processed images were analyzed for fluorescence intensity using the ImageJ software (NIH).

**Table 2:** List of antibodies for immunofluorescence staining.

Antibody	Host	Company	Catalogue number
anti-human CD29	mouse	Millipore	MAB2253Z
anti-human Flk-1	mouse	Santa Cruz	Sc-6251
anti-human CD34	mouse	Millipore	MAB4211
anti-human CD31	mouse	Dako	M0823
anti-human CD31	rabbit	abcam	ab76533
anti-human CD45	mouse	abcam	ab82595
anti-human CD90	mouse	BD biosciences	555595
anti-human CD105	mouse	Caltac Laboratories	MHCD10500
anti-human αSMA	mouse	Dako	M0851
anti-Caspase 3	rabbit	abcam	ab4051
anti-human nuclei	mouse	Millipore	MAB1281
HIF-1 alpha	rabbit	Novus	NB100-134
anti-human FGF	rabbit	abcam	ab8880
anti-human VEGF	rabbit	abcam	ab52917
anti-human HGF	rabbit	Santa Cruz	Sc-13087
Alexa Fluor 488 anti-mouse IgG	goat	Invitrogen	A11001
Alexa Fluor 594 anti-rabbit IgG	goat	Invitrogen	A11012

### Histological staining

Samples were harvested 14 days after treatment. Specimens were fixed in 10% (v/v) buffered formaldehyde, dehydrated in a graded ethanol series, and embedded in paraffin. Specimens were sliced into 4 μm-thick sections and were stained with hematoxylin and eosin (H&E) to examine muscle degeneration and tissue inflammation. Masson’s trichrome collagen staining was performed to assess tissue fibrosisin ischemic regions. The criteria used for the histological scores of wound healing were modified from previous reports [17] and are summarized in Table 3. The histological parameters considered were reepithelialization, dermal regeneration, granulation tissue formation, and angiogenesis. Regeneration of skin appendages was assessed by counting the number of hair follicles or sebaceous glands in the wound bed.

**Table 3:** Histological scoring system.

Scores	Re-epithelializtion	Dermal regeneration	Granulation tissue formation	Angiogenesis
1	Minimal epidermal regeneration (<50%)	No skin appendage formation	Thin granulation around wound edges only	Little angiogenesis (<10 vessels/HPF)
2	Moderate epidermal regeneration (50%)	A few skin appendage formation (<5 appendages/ wound area)	Moderate granulation in the wound bed	Moderate angiogenesis (10–20 vessels/HPF)
3	Complete epidermal regeneration (100%)	Considerable skin appendage formation (>5 appendages/ wound area)	Thick granulation in 100% of the wound bed	Marked newly formed and well-structured capillary vessels (>20 vessels/HPF)

### Western blot analysis

Samples were solubilized in lysis buffer [20 mM Tris-HCl, pH 7.4, 150 mM NaCl, 1 mM EDTA, 1% Triton X-100, 0.1% sodium dodecyl sulfate (SDS), 1 mM phenylmethylsulfonyl fluoride, 1μg/ml leupeptin, and 2 μg/ml aprotinin] for 1 h at 4°C. The lysates were then clarified by centrifugation at 15,000 g for 30 min at 4°C, were diluted in Laemmli sample buffer containing 2% SDS and 5% (v/v) 2-mercaptoethanol, and were heated for 5 min at 90°C. The proteins were separated via SDS polyacrylamide gel electrophoresis (PAGE) using 10% or 15% resolving gels followed by transfer to nitrocellulose membranes (Bio-Rad, Hercules, CA) and then probed with antibodies against HIF-1a (Novus), CD31 (Abcam), HGF (Santa cruz), VEGF (Abcam), and FGF2 (Abcam) for 1h at room temperature (Table 2). Peroxidase-conjugated anti-mouse IgG or anti-rabbit IgG and enhanced chemiluminescence (Amersham Pharmacia Biotech, Piscataway, NJ) were used as described by the manufacturer for detection. The membranes were scanned to create chemiluminescent images that were then quantified with an image analyzer (Kodak).

### Preparation of the experimental animal model

The animal studies were approved by the Dankook University Animal Use and Care Committee. Five-week-old male BALB/c nude mice (20 g body weight; Narabio, Seoul, Korea) were anesthetized with ketamine (100 mg/kg). After aseptically preparing the surgical site, two full-thickness skin wounds were created on the dorsal part using an 8-mm biopsy punch. To inhibit wound contraction, a 0.5-mm thickness silicone splint was applied, as has been previously described [18]. The splint was fixed with instant adhesive and six simple, interrupted sutures around the wound with Nylon 6–0. The wounds were randomly classified into five groups: control (*n* = 9), LLLT (*n* = 9), ASCs (15 × 10^5^ cells; hASCs group, *n* = 9), spheroid (10 masses; spheroids group, *n* = 9), and spheroid + LLLT (10 masses; spheroids + LLLT group, *n* = 9). In the ASCs, spheroid, and spheroid + LLLT groups, 15 × 10^5^ ASCs in 100 μl of PBS were transplanted intradermally at four injection sites on the border between the wound and the normal skin. The control group received a PBS injection of PBS (PBS group, *n* = 9). The physiological status of the wound was followed up for up to 2 weeks after treatment. Tegaderm (3M Health Care, MN, USA) was used for wound protection, and an equivalent number of cells were injected in both conditions.

### Low-level light therapy in skin

Light emitting diode (LED; WON Technology, Daejeon, Korea) was applied for 10 min daily from day 1 to 20. The distance from the LED to the skin flap was 8 cm. This LED model exhibited an irradiated wavelength of 660 nm and power density of 50 mW/cm^2^. The fluence of each flap site was 30 J/cm^2^ (1 milliwatt × second = 0.001 joules) (Table 1).

### Gross evaluation of the wound area

The wounds were photographed using a digital camera at 3, 7, and 14 days after surgery, and the wound area was measured by tracing the wound margin and then performing the calculation using the Image J image analysis program (NIH, MD, USA). The wound area was analyzed by calculating the percentage of the current wound with respect to the original wound area. The wound was considered to be completely closed when the wound area was grossly equal to zero.

### Statistical analyses

All quantitative results were obtained from triplicate samples. Data were expressed as a mean ± SD, and the statistical analyses were carried out using two-sample t tests to compared two groups of samples and a One-way Analysis of Variance (ANOVA) for the three groups. A value of p < 0.05 was considered to be statistically significant.

## Results

### Characterization of hASCs

hASCs obtained from human adipose tissue were expanded *in vitro*. The cells were positive for human MSC markers CD29 (β1 integrin), CD90 (Thy-1) and CD105 (endoglin). However, the cells were found to be negative for human endothelial cell markers CD34, CD31, and KDR (VEGF receptor) through immunofluorescent staining and flow cytometry analyses (S1A and S1B Fig). These results indicated that the expanded cells included a large population of hASCs and were not contaminated with endothelial cells (EC).

### Effect of LLLI on the migration and survival of hASCs

A wound scratch test showed that the migration of LLLI-treated hASCs markedly improved relative to that of control cells cultured without LLLI after 24 hours (S2 Fig). There were statistically significant differences between the experimental and the control groups (*p < 0*.*05*). To verify the cell viability of the L-spheroid, a live/dead assay with of fluorescent dyes was carried out (S3 Fig). Non-viable cells were stained red, and viable cells were stained green. Apoptosis induced by a lack of cell-matrix interaction (anoikis) was prevented in hASCs cultured as L-spheroids.

### Production of Angiogenic Factors by hASCs in L-spheroids hASCs

**Fig 1:**
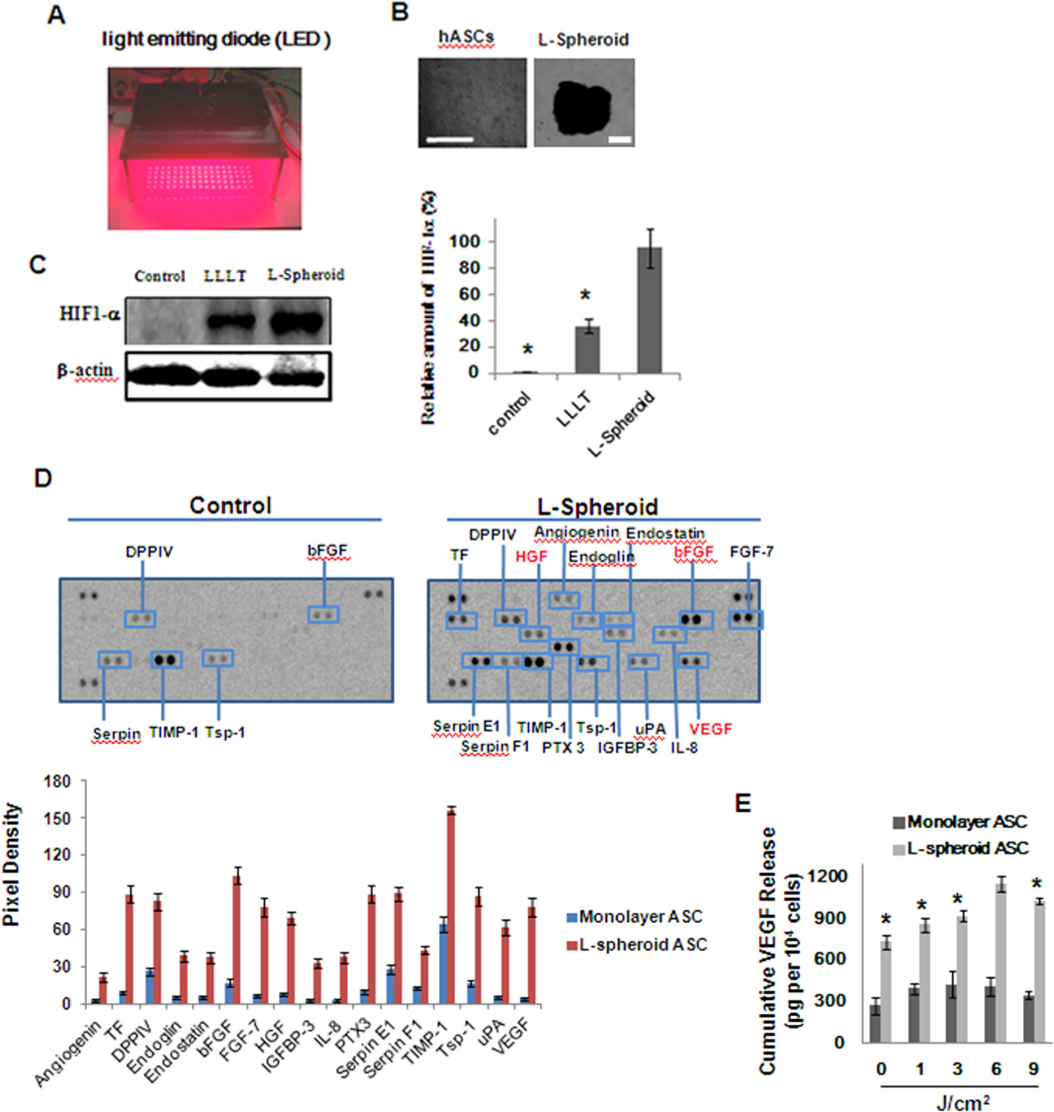
Enhanced expression of hypoxia-induced survival factors and angiogenic growth factors in hASC L-spheroids. (A) The light source used was LED (660 nm) designed to fit over a microplate (12.5 × 8.5 cm) for cell culture. (B) Formation of hASC L-spheroids. hASCs morphology on non–tissue culture–treated 24-well plates at day 3. Scale bar = 500 μm. (C) Western blot analysis and quantification of HIF1-α in hASCs cultured as spheroids, L-spheroids and monolayers (*p < 0.*01*, compared to the L-spheroid group). (D) Angiogenesis-related protein analysis of L-spheroids (*, *p* < 0.05, compared to the spheroid group, t-test, *n = 3* in each group). (E) ELISA measurement of spheroids cultured for 3 days. Concentrations of VEGF are presented as pg-corrected for 10^4^ cells. (*, *p* < 0.05, compared with spheroid 6J/cm^2^ group, t-test, n = 3 in each group).

hASCs were cultured on non-tissue culture-treated 24-well plates in the presence of FBS and formed a floating spheroid after irradiation with low-level light (Fig 1A) 3 days after seeding. The diameter of most L-spheroids ranged from 1.2 to 1.5 mm (Fig 1B). L-spheroid cultures showed a dramatic increase in the expression of hypoxia-induced survival factors, such as hypoxia-inducible factor (HIF)-1α, relative to cells in a monolayer culture (Fig 1C). Therefore, L-spheroid hASCs seemed to be more adaptable and more resistant to hypoxia compared to hASCs in monolayer cultures. HIF-1α is known to upregulate the expression of angiogenic growth factors [19, 20], and L-spheroid hASCs showed considerable expression of angiogenic growth factors, *i*.*e*., hepatocyte growth factor (HGF), vascular endothelial growth factor (VEGF), and fibroblast growth factor 2 (FGF2). The expression of angiogenic growth factors in L-spheroid hASCs was much greater than that of hASCs cultured in a monolayer culture (Fig 1D and 1E).

### Endothelial Cell Differentiation of hASC L-spheroid hASCs

**Fig 2:**
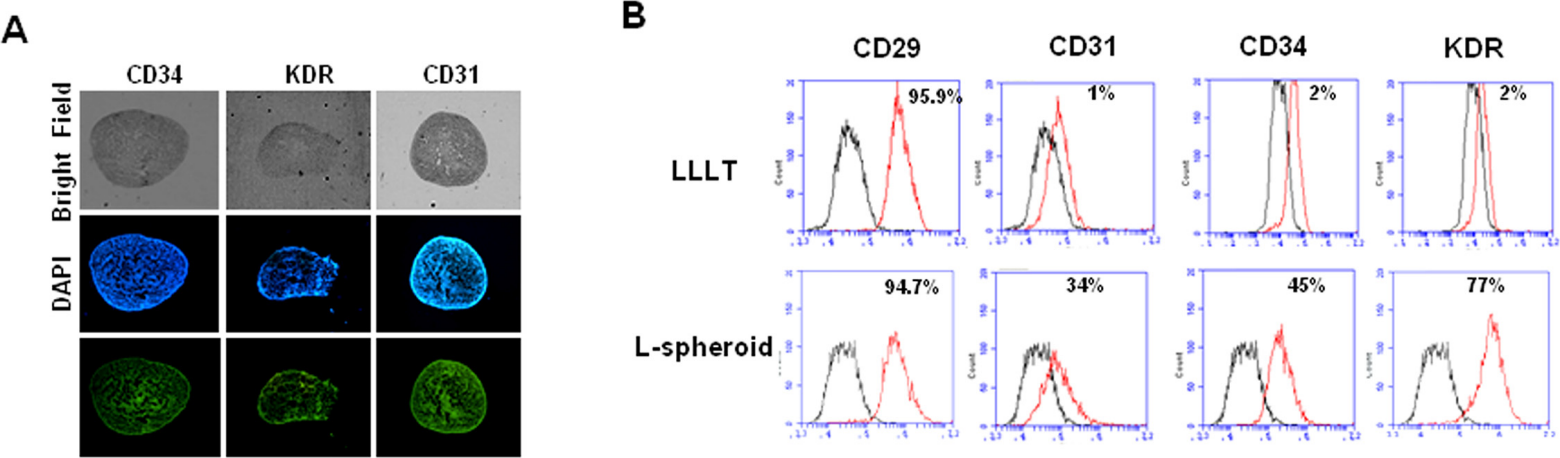
Endothelial phenotyping of L-spheroids. (A) Monitoring cell surface markers via immunofluorescence staining. L-spheroids cultured for 3 days were cryosectioned and stained with anti human CD34, CD31, and KDR antibodies. Scale bar = 500 μm. (B) Flow cytometry analysis.

The endothelial phenotype of the L-spheroid cells was also evaluated via immunofluorescent staining for a variety of endothelial cell surface markers. First, the surface markers of hASCs expanded in DMEM-F12/FBS were examined. The cells expressed CD29 (β1 integrin), CD90 (Thy-1), and CD105 (endoglin), as well as MSC surface antigens, but not CD34, CD31, or KDR (S2B and S2C Fig). This indicated that the cells used in the study included a large population of hASCs without endothelial lineage cell contamination. Immunofluorescence staining also revealed that L-spheroids expressed a variety of EC surface markers, including CD34, CD31, and KDR (VEGF receptor) (Fig 1B). The cell population of the L-spheroids was further characterized via flow cytometry. In a monolayer culture with LLLI, EC markers (CD34, CD31, and KDR) were detected in less than 1% of cells. Conversely, L-spheroids were composed of a population of cells positive for CD34, CD31, and KDR (Fig 2B).

### Survival of ASCs in the wound bed

**Fig 3:**
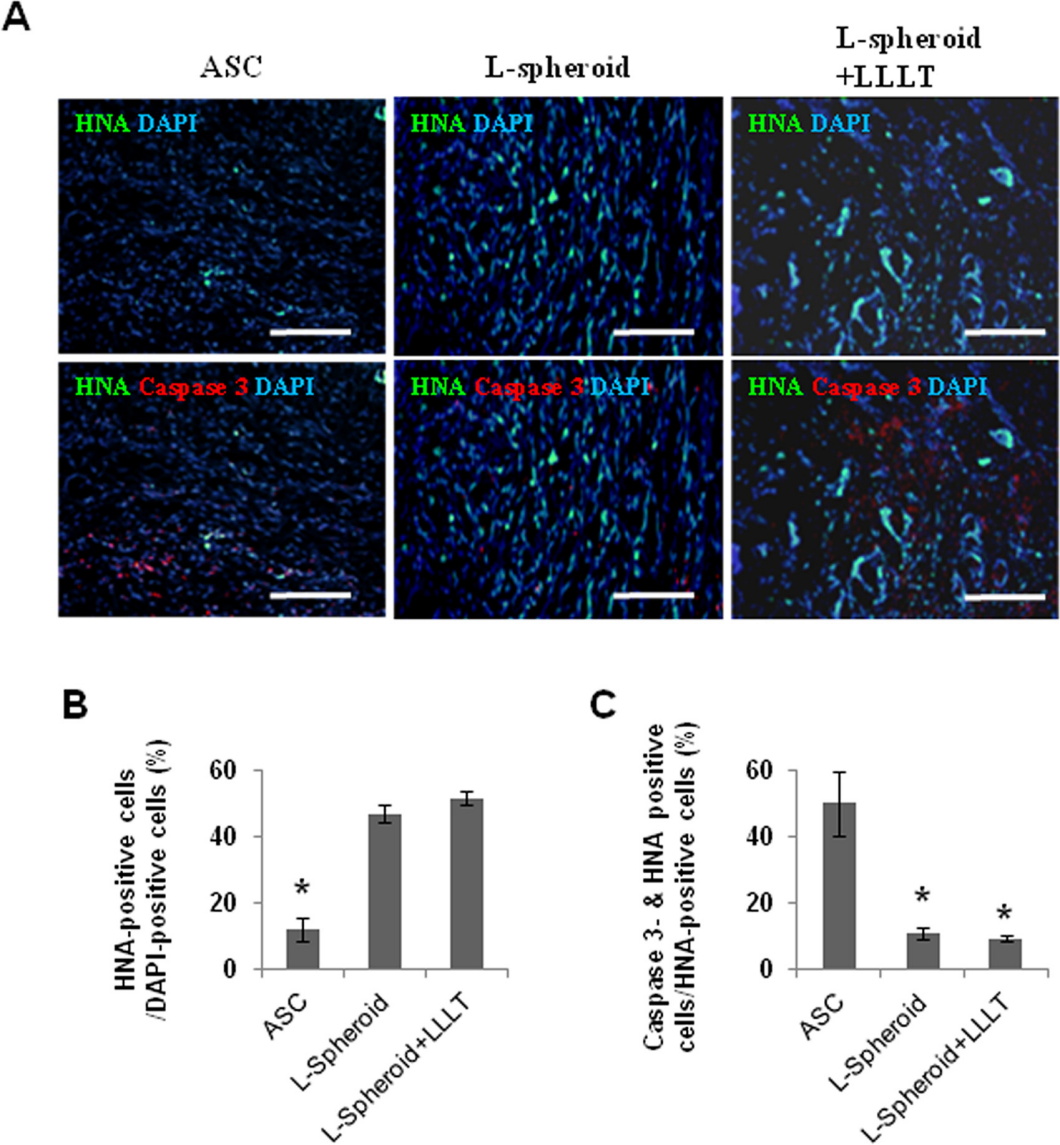
Survival of transplanted hASCs in the wound bed. (A) For the ASCs, L-spheroid and L-spheroid + LLLT groups, DAPI (blue) and caspase 3 (apoptotic marker; red)-positive cells were detected after immunostaining at 14 days. The hASCs were stained with HNA (green). Apoptosis of transplanted hASCs (arrows) was reduced in the L-spheroid + LLLT group. (B) The ratio of HNA-positive cells (transplanted hASCs) to DAPI-positive cells (total cells) in the wound bed (*p < 0.*01*). (C) Ratio of caspase-3-positive cells plus HNA-positive cells (apoptotic transplanted hASCs) to HNA-positive cells (transplanted hASCs) in the wound bed (*p < 0.*01*).

After 14 days, fluorescence microscopy was used to identify caspase 3-positive cells and HNA-positive cells throughout the wound bed to determine whether locally transplanted ASCs were incorporated into the healing wound. In the L-spheroid and L-spheroid + LLLT groups, ASCs were observed in the regenerated skin tissue (Fig 3A). The L-spheroid + LLLT group exhibited significantly increased numbers of HNA-positive cells (ASC group: 11%; L-spheroid group: 46%; L-spheroid + LLLT group: 51% per DAPI-positive cells) (Fig 3B) and decreased proportions of caspase 3-positive ASCs (ASC group: 36%; L-spheroid group: 12%; L-spheroid + LLLT group: 9% per HNA-positive cells) (Fig 3C). The HNA+ cell per DAPI+ cell ratio of the L-spheroid + LLLT group was 4.6 times higher than that of the hASC group.

### Enhanced secretion of growth factors from grafted hASCs in the wound bed

**Fig 4:**
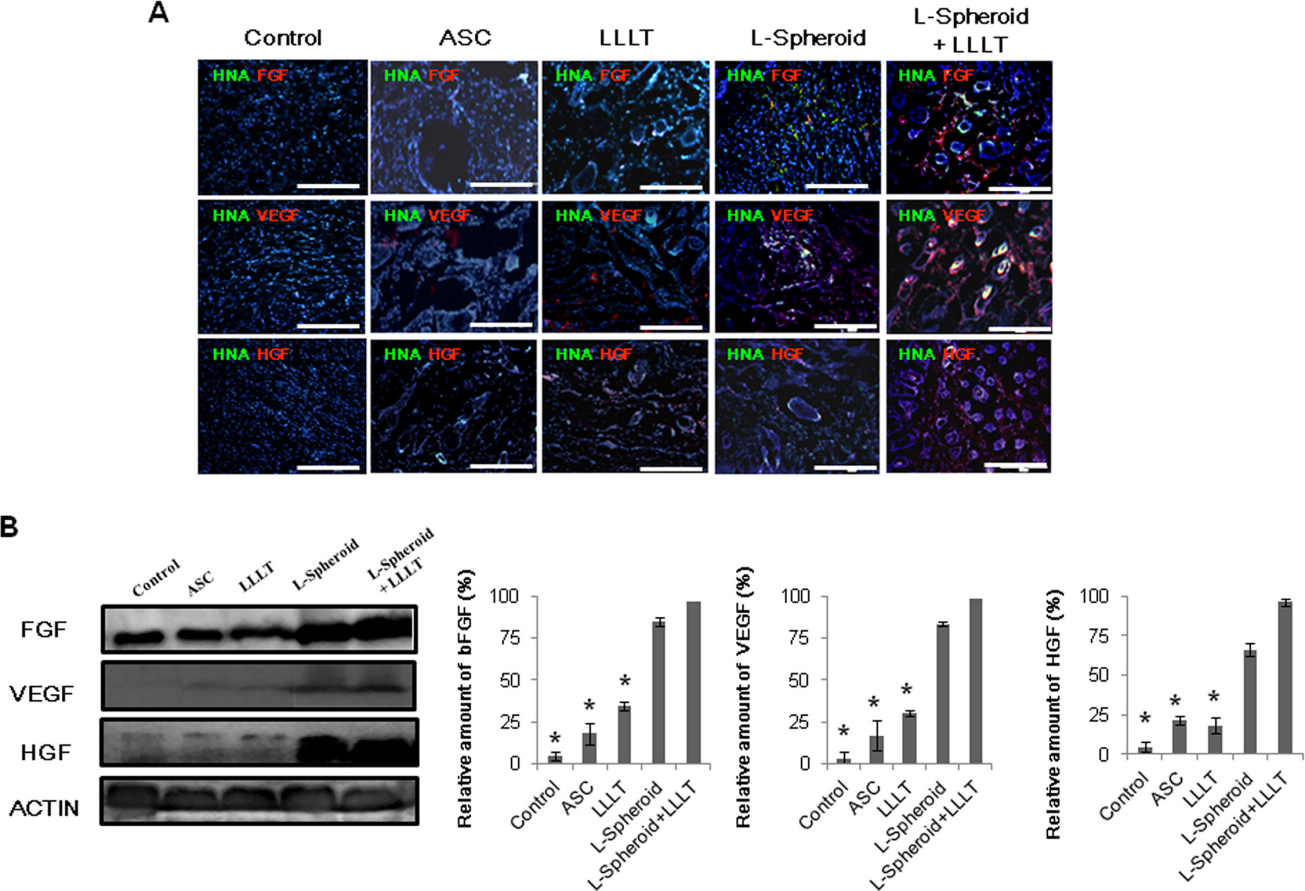
Enhanced secretion of angiogenic growth factors from hASCs in the wound bed. (A) Immunostaining was performed with anti-bFGF and anti-VEGF or anti-HGF antibody (red) at 14 days. The scale bar indicates 100 μm. (B) Western blot indicated the expression of bFGF, VEGF, and HGF at 14 days. The results of the Western blot were analyzed as relative density (*p < 0.*05*, compared to the spheroid + LLLT group).

Transplantation of hASCs into the wound bed enhanced the paracrine secretion of angiogenic growth factors. Double immunofluorescent staining of HNA and human angiogenic growth factors bFGF, VEGF, and HGF indicated the presence of secretion from transplanted hASCs in the ASC or spheroid group (Fig 4A). Secreted human growth factors were mainly distributed in the vicinity of transplanted hASCs (HNA-positive cells), and as compared to the ASC group, more growth factor-positive ASCs were observed in the L-spheroid + LLLT group (Fig 4A). A Western blot assay showed that significantly higher levels of VEGF, bFGF, and HGF were secreted by the L-spheroid and L-spheroid + LLLT groups than by the control group, and greater amounts of growth factors were observed in the L-spheroid + LLLT group than in the ASC group (Fig 4B). However, no significant difference was observed between the ASC-treated tissues and the control tissues, indicating that the L-spheroid groups were more effective than the ASC group at increasing transplanted cell retention and angiogenic growth factor expression.

### Angiogenic efficacy in the wound bed

**Fig 5:**
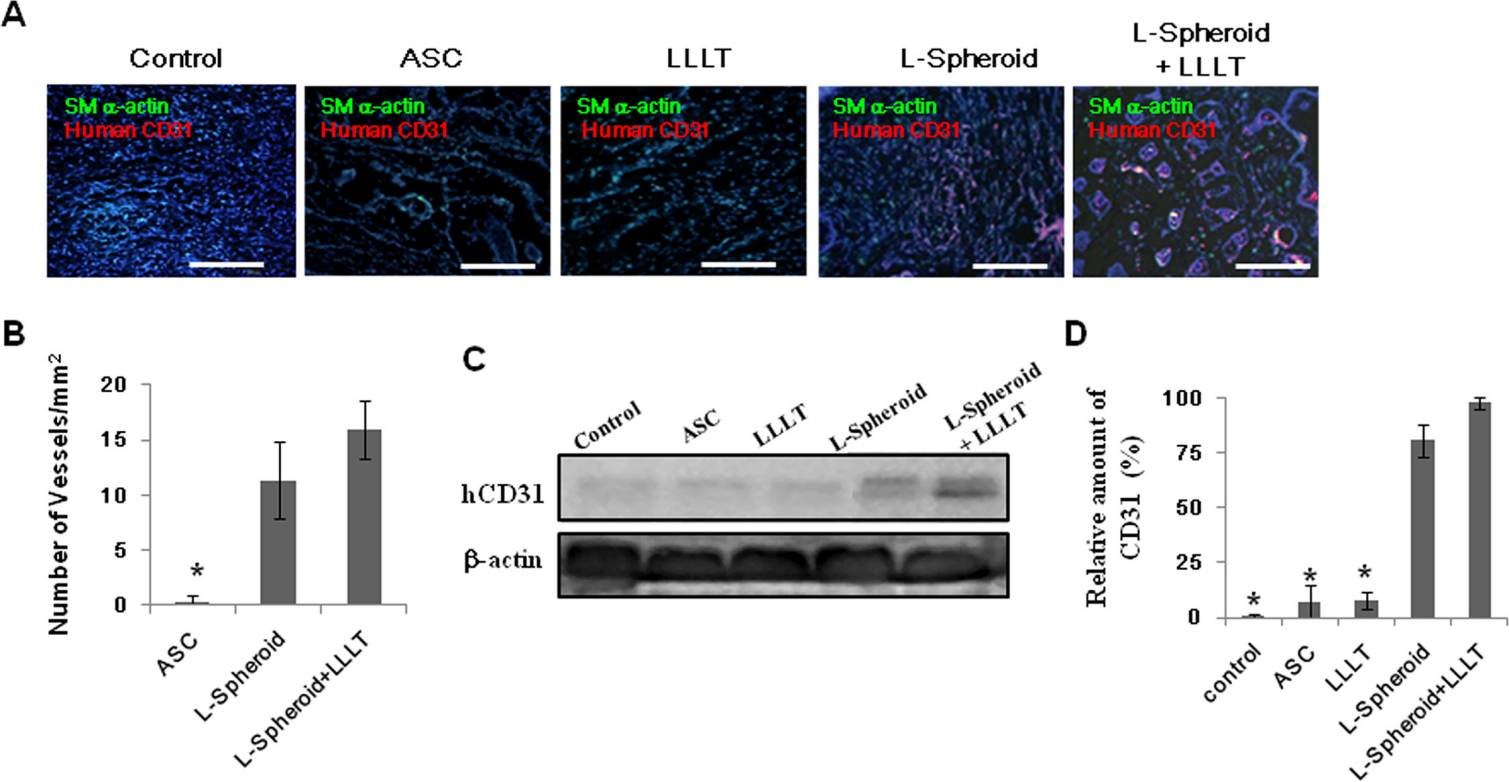
Endothelial cell and smooth muscle cell differentiation of transplanted cells. (A) The implants were removed on day 14 after transplantation and were stained with anti human CD31 and human αSMA antibody. The scale bars indicate 200 μm. (B) Vessel density in the wound bed (*p < 0.*05* compared to the spheroid + LLLT group). (C) Western blot indicates the expression of HIF-1α and CD31 at 14 days. (D) Western blot analysis quantification (*p < 0.*01*, compared to the spheroid + LLLT group).

Many of the CD31+ cells in the L-spheroid + LLLT group were double stained for smooth muscle actin (SMA). ECs and perivascular cells differentiated from injected human cells were detected via αSMA and human CD31 antibodies, respectively (Fig 5A and 5B). A Western blot assay presented significantly higher levels of CD31 secreted by the L-spheroid and L-spheroid + LLLT groups than by the control group (Fig 5C and 5D) and greater amounts of growth factors in the L-spheroid + LLLT group than in the ASC group (Fig 4D). However, there was no significant difference between the ASC-treated tissues and the control tissues. These findings suggest the greater effectiveness of the L-spheroid + LLLT treatment for angiogenesis in the wound bed.

### Differentiation of ASCs into epithelial cells

**Fig 6:**
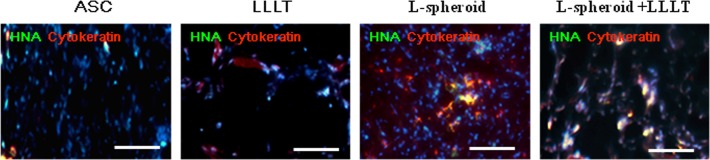
Differentiation of ASCs into epithelial cells. Immunofluorescence images show cytokeratin-positive epithelial cells (red) at 14 days. The scale bar indicates 20 μm.

To determine whether the L-spheroid ASCs could contribute to the epidermal structure, immunohistochemistry for pan-cytokeratin was performed at 14 days (Fig 6). Some cytokeratin-positive ASCs were found in the epidermis or the sebaceous glands in the spheroid and L-spheroid + LLLT group.

### Wound closure and dermal reaction

**Fig 7:**
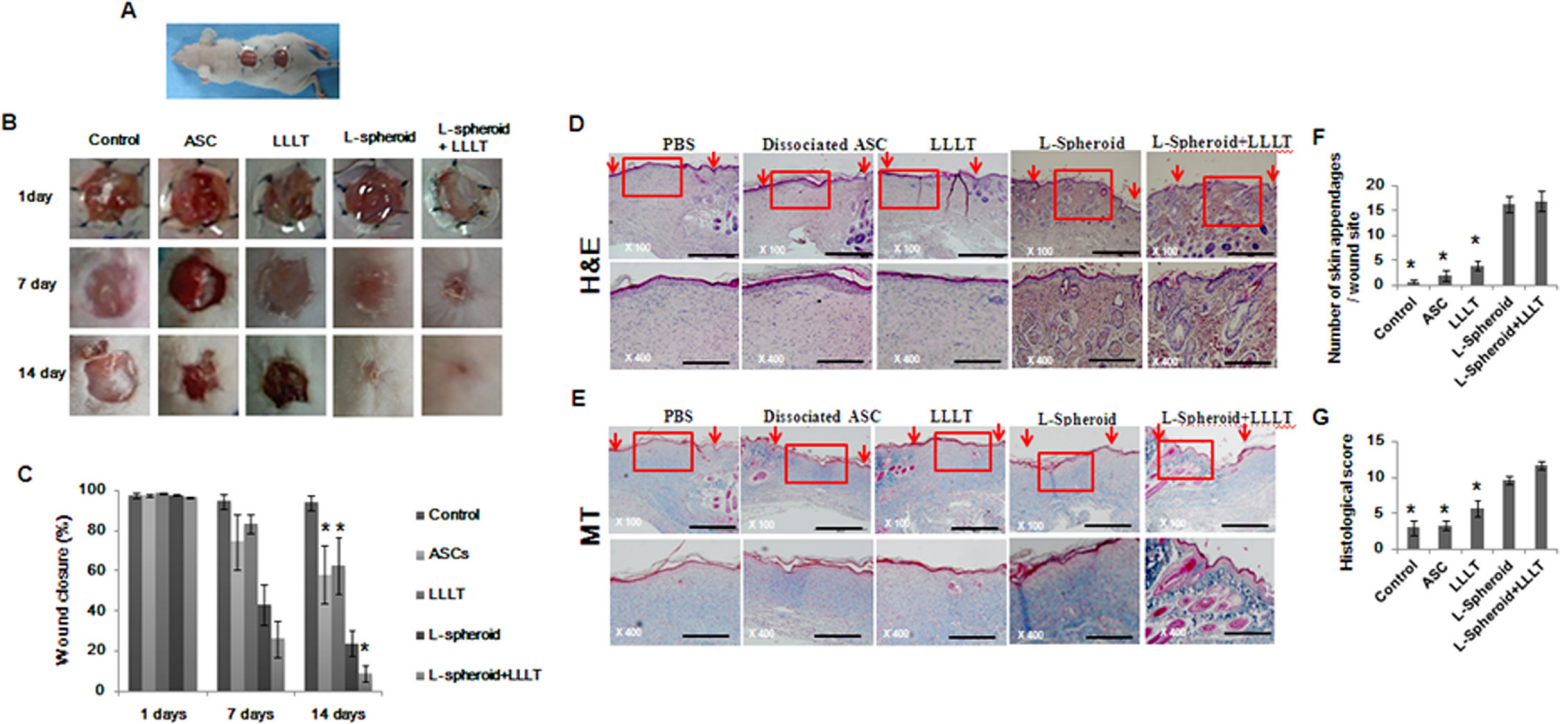
Evaluation of the wound closure. (A) The prepared excisional wound splinting model. (B) Photographs of the wounds. (C) The percentage of the wound area was calculated using photographs of the wounds at 1, 7, and 14 days. *p < 0.05 versus the L-spheroid group. (D, E) Histological analysis of the wound bed. Wounds were stained with (D) H&E and (E) Masson’s trichrome at 14 days. The wound edges are indicated with arrowheads. The closed arrows indicate skin appendages (hair follicles). The scale bar indicates 500 μm. (F) The regeneration of skin appendages was investigated by counting the number of skin appendages per wound section. (G) Histological scoring was performed using the criteria presented in Table 3. *p < 0.05, versus the L-spheroid group.

An excisional wound splinting model was prepared, and the silicon splints remained tightly adherent to the skin and restricted wound contraction during the experimental period (Fig 7A). At 7 and 14 days after the surgery, the L-spheroid and L-spheroid + LLLT groups exhibited significantly smaller wound areas than did the other groups. At 7 days, the L-spheroid + LLLT group showed a significantly smaller wound area than the ASCs and the L-spheroid groups did. No significant difference was observed between control and LLLT group at any time (Fig 7B and 7C). At 14 days, all of the wounds of the L-spheroid and L-spheroid + LLLT groups achieved complete closure, but not all of the wounds of the control and LLLT groups had completely closed. The histological observation showed that skin regeneration was much greater in the L-spheroid and L-spheroid + LLLT groups compared to the control group. Our data indicated that the L-spheroid enhanced re-epithelialization and granulation at 14 days (Fig 7D). Furthermore, the L-spheroid groups appeared to have an increased number of skin appendages (Fig 7F and 7G). The L-spheroid groups displayed significantly increased numbers of hair follicles and sebaceous glands 14 days (Fig 7D–7G).

## Discussion

The formation of spheroids is affected by the cell-matrix adhesion strength [21]. Moreover, LLLI can promote the migration of hASCs [9, 22, 23]. In this study, within 3 days of culture on non–tissue culture–treated 24-well plates, hASCs formed spheroids as a result of low-level light irradiation (L-spheroids) (Fig 1A). Notably, an increased HIF-1α expression and the consequent induction of protein for VEGF and FGF occurred at a fluence of 660 nm. It had been previously reported that hypoxia mediates the angiogenic switch in agglomerates of tumor cells larger than 200 μm in diameter [12]. To confirm that a hypoxic environment developed in the spheroid cultures of the hASCs, we detected protein expression of HIF-1α (Fig 1B). HIF-1α expression is primarily induced by hypoxia, but its induction can also be mediated by growth factors and cytokines [9, 22, 23]. This protein is stabilized at low oxygen tensions while at higher oxygen tensions it is rapidly degraded by oxygen-dependent prolyl hydroxylase enzymes. HIF-1α regulates the cellular response to physiological and pathological hypoxia by activating genes that are important to cellular adaptation and survival pathways under hypoxic conditions [12, 24, 25]. In our experimental model, we observed that the non-irradiated group expresses HIF-1α in response to the hypoxic environment formed in the hASC spheroid. In the irradiated groups, 660-nm light alone is able to increase HIF-1α expression in this model regardless of the fluence used (Fig 1B). In this case, induction does not depend on oxygen tension and involves the activation of a different regulatory mechanism, possibly mediated by mitogen-activated protein kinase and the phosphatidylinositol 3-kinase/Akt signaling pathway [26]. Oxidative stress can also increase the expression of this transcription factor in the spheroid. Numerous studies have reported that hypoxia induces the production of growth factors correlated with endothelial cell growth and function [27]. In practice, hASCs can differentiate in vitro into functional endothelial cells in the presence of angiogenic factors such as 50 ng/mlVEGF [28]. Therefore, it is expected that the endothelial differentiation of hASCs in the spheroid might be up-regulated by angiogenic factors, such as VEGF (Fig 1C and 1D). In two-dimensional cultures, growth factors secreted from the cells are released and diluted in the culture supernatant, preventing cells from responding to the released factors. Conversely, in L-spheroid cultures, if growth factors are secreted from stem cells after 3D cell aggregation, the factors might be stored in the cell spheroid and may then stimulate endothelial differentiation of the stem cells (Fig 2).

Clinical studies of stem cell transplantation have raised several questions concerning cell therapy. The density and complexity of vascular networks formed by the synergistic dual cell system were reported to be was many times greater than those observed with ASC- containing and EC- or SMC-containing implants [29]. In addition, gel-assisted subcutaneous injection of VEGF- and bFGF-expressing ECs formed mature vasculature, whereas those expressing VEGF did not form mature vasculature. In this study, L-spheroid ASCs accelerated wound closure with an increased level of re-epithelialization, neovascularization, and regeneration of skin appendages. This is likely to be due to the enhanced survival of L-spheroid hASCs along with increased paracrine secretion (Fig 3). Our data revealed the presence of an increased number of ASCs and a decreased percentage of caspase 3-positive ASCs at 14 days in the L-spheroid group relative to the ASCs group. These data suggest that LLLI enhanced the survival of the spheroid ASCs by inhibiting apoptosis. In addition, VEGF, bFGF, and HGF positive-ASCs were detected in the wound bed (Fig 4). VEGF is the most effective and specific growth factor that regulates angiogenesis [30]; bFGF is an important growth factor in wound healing because it affects the migration and proliferation of fibroblasts, angiogenesis, and matrix deposition [31]; and HGF is another potent proangiogenic factor that induces migration and proliferation and inhibits apoptotic cell death of ECs [32]. The L-spheroid complex appeared to promote vasculogenesis through a synergistic effect in the wound bed (Fig 5). It is possible that LLLI enhances cellular responses in terms of gene expression, secretion of growth factors, and cell proliferation through an increase in the mitochondrial membrane potential and the ATP and cAMP levels [26]. In addition to the sebaceous glands, some cytokeratin positive-ASCs were observed in the regenerated epidermis (Fig 6). Several recent studies that reported ASCs to enhance wound repair as a result of differentiation and of their paracrine effects are consistent with the results of our study. For example, Smith et al (2010) reported that MSCs migrate into the wound area [33] and differentiate into keratinocytes, endothelial cells, sweat glands, sebaceous glands, and hair follicles [17, 34]. Additionally, Wu et al (2007) have shown that MSCs secrete paracrine factors, such as VEGF, bFGF, epidermal growth factor, keratinocyte growth factor, insulin-like growth factor, and hepatocyte growth factor, and stimulate the deposition of extracellular matrix [17, 35]. In this study, the L-spheroid groups were found to exhibit rapid wound closure and a higher histological score relative to the ASCs group (Fig 7). In skin bioengineering, the ultimate goal is to rapidly produce a construct that offers complete restoration of functional skin, ideally involving the regeneration of all skin appendages and layers [2]. Interestingly, our results showed that the L-spheroid groups present significantly increased numbers of sebaceous glands than the ASCs group. These results suggest that L-spheroid ASCs enhanced not only survival, but also the functionality of the transplanted ASCs in the wound bed.

## Conclusions

hASC L-spheroids transplantation accelerates tissue regeneration through the differentiation of ECs and through growth factor secretion. We emphasize the significance of the application of a 3D spheroid culture of stem cells with LLLI to achieve a high-ratio of EC differentiation of hASCs and to enhance treatment efficiency of L-spheroid transplantation relative to single-cell transplantation in the wound bed. These results may provide more effective therapeutic methods to treat delayed skin regeneration.

## Supporting Information

S1 Fig Immunofluorescence staining and flow cytometry analyses of hASCs. hASCs (passage 4) were stained with CD29, CD90 and CD105 for mesenchymal stem cell identification, with KDR, CD31 and CD34 for endothelial lineage cell identification, and SMA for smooth muscle cell identification. Scale bar: 200 μm (B) Flow cytometry analysis; hASCs cultured for 1 days were stained for CD29, CD90, CD105, CD45, CD31, CD34 and KDR expression and analyzed by flow cytometry.
(TIF)

S2 Fig Migration of BMSCs by wound scratch test. LLLI treated hASCs scratch wound at 24 h (*, *p* < 0.05, compared with LLLT group, t-test, *n = 3* in each group).
(TIF)

S3 Fig Fluorescence microscopic image of Live/Dead stain on day 1. The middle-section was 500 μm from the L-spheroid surface. Live cells were stained by calcein AM (green), and dead ones were stained with ethidium homodimer (red). Scale bar: 500 μm.
(TIF)
