# Supplementary figures and images for: Enhancement of Ischemic Wound Healing by Spheroid Grafting of Human Adipose-Derived Stem Cells Treated with Low-Level Light Irradiation
Source: PLoS One. 2015 Jun 11;10(6):e0122776. doi: 10.1371/journal.pone.0122776 (PMC4465903; doi:10.1371/journal.pone.0122776)

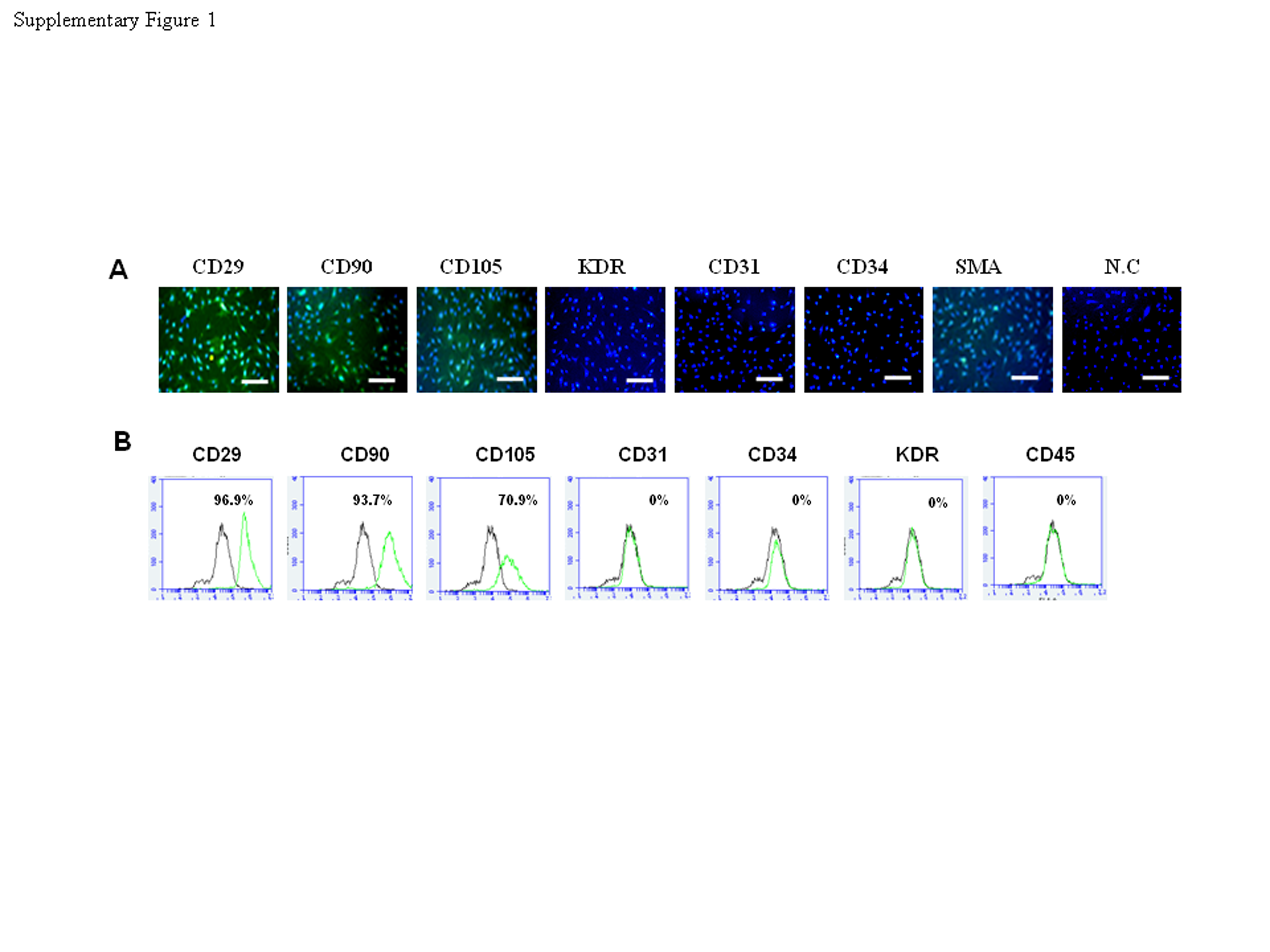

Supplement: S1 Fig — hASCs (passage 4) were stained with CD29, CD90 and CD105 for mesenchymal stem cell identification, with KDR, CD31 and CD34 for endothelial lineage cell identification, and SMA for smooth muscle cell identification. Scale bar: 200 μm (B) Flow cytometry analysis; hASCs cultured for 1 days were stained for CD29, CD90, CD105, CD45, CD31, CD34 and KDR expression and analyzed by flow cytometry. (TIF) [file pone.0122776.s001.TIF]

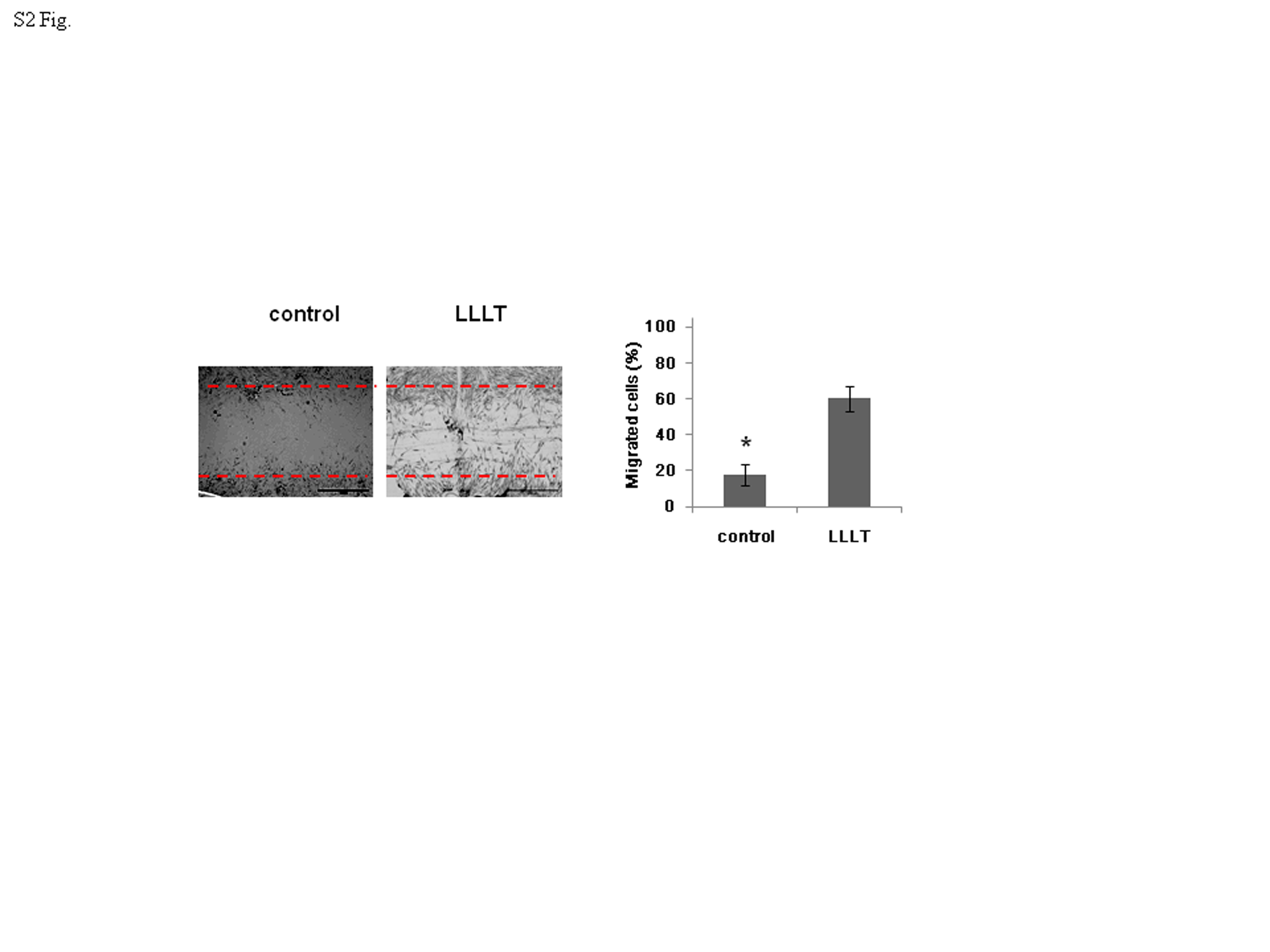

Supplement: S2 Fig — LLLI treated hASCs scratch wound at 24 h (*, p < 0.05, compared with LLLT group, t-test, n = 3 in each group). (TIF) [file pone.0122776.s002.TIF]

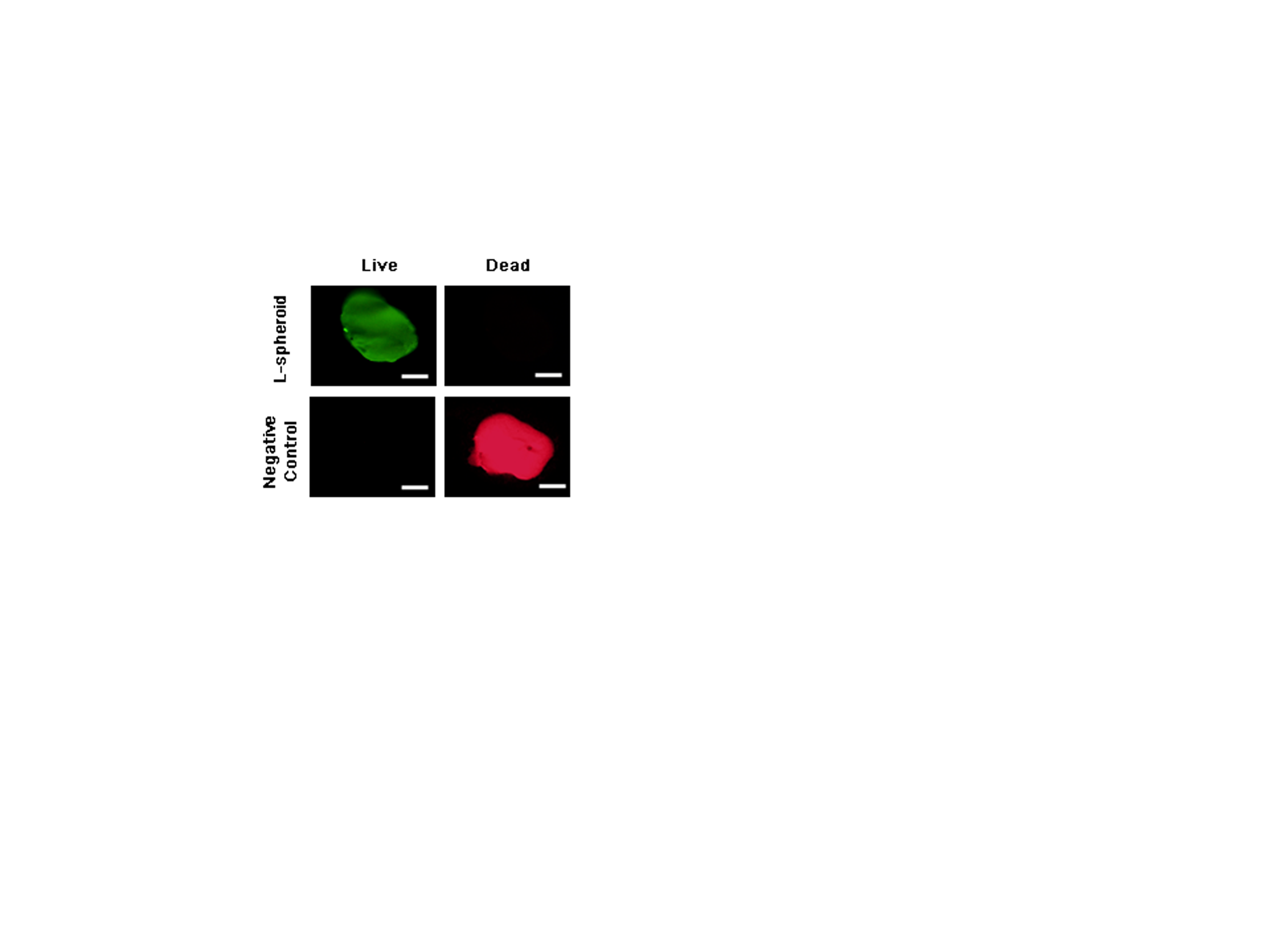

Supplement: S3 Fig — The middle-section was 500 μm from the L-spheroid surface. Live cells were stained by calcein AM (green), and dead ones were stained with ethidium homodimer (red). Scale bar: 500 μm. (TIF) [file pone.0122776.s003.TIF]
